# Ceftolozane/tazobactam disrupts *Pseudomonas aeruginosa* biofilms under static and dynamic conditions

**DOI:** 10.1093/jac/dkae413

**Published:** 2024-12-09

**Authors:** Xenia Kostoulias, Ying Fu, Faye C Morris, Crystal Yu, Yue Qu, Christina C Chang, Luke Blakeway, Cornelia B Landersdorfer, Iain J Abbott, Lynn Wang, Jessica Wisniewski, Yunsong Yu, Jian Li, Anton Y Peleg

**Affiliations:** Infection Program, Department of Microbiology, Monash Biomedicine Discovery Institute, Monash University, Melbourne, VIC 3800, Australia; Department of Infectious Diseases, The Alfred Hospital and School of Translational Medicine, Monash University, Melbourne, VIC 3004, Australia; Centre to Impact AMR, Monash University, Melbourne, VIC 3800, Australia; Infection Program, Department of Microbiology, Monash Biomedicine Discovery Institute, Monash University, Melbourne, VIC 3800, Australia; Department of Clinical Laboratory, Sir Run Run Shaw Hospital, College of Medicine, Zhejiang University, Hangzhou, Zhejiang, China; Infection Program, Department of Microbiology, Monash Biomedicine Discovery Institute, Monash University, Melbourne, VIC 3800, Australia; Centre to Impact AMR, Monash University, Melbourne, VIC 3800, Australia; Infection Program, Department of Microbiology, Monash Biomedicine Discovery Institute, Monash University, Melbourne, VIC 3800, Australia; Infection Program, Department of Microbiology, Monash Biomedicine Discovery Institute, Monash University, Melbourne, VIC 3800, Australia; Department of Infectious Diseases, The Alfred Hospital and School of Translational Medicine, Monash University, Melbourne, VIC 3004, Australia; Centre to Impact AMR, Monash University, Melbourne, VIC 3800, Australia; Department of Infectious Diseases, The Alfred Hospital and School of Translational Medicine, Monash University, Melbourne, VIC 3004, Australia; Department of Infectious Diseases, The Alfred Hospital and School of Translational Medicine, Monash University, Melbourne, VIC 3004, Australia; Centre to Impact AMR, Monash University, Melbourne, VIC 3800, Australia; Drug Delivery, Disposition and Dynamics, Monash Institute of Pharmaceutical Sciences, Monash University, Melbourne, VIC 3052, Australia; Department of Infectious Diseases, The Alfred Hospital and School of Translational Medicine, Monash University, Melbourne, VIC 3004, Australia; Microbiology Unit, The Alfred Hospital, Prahran, Melbourne, VIC 3004, Australia; Infection Program, Department of Microbiology, Monash Biomedicine Discovery Institute, Monash University, Melbourne, VIC 3800, Australia; Department of Infectious Diseases, The Alfred Hospital and School of Translational Medicine, Monash University, Melbourne, VIC 3004, Australia; Department of Clinical Laboratory, Sir Run Run Shaw Hospital, College of Medicine, Zhejiang University, Hangzhou, Zhejiang, China; Infection Program, Department of Microbiology, Monash Biomedicine Discovery Institute, Monash University, Melbourne, VIC 3800, Australia; Infection Program, Department of Microbiology, Monash Biomedicine Discovery Institute, Monash University, Melbourne, VIC 3800, Australia; Department of Infectious Diseases, The Alfred Hospital and School of Translational Medicine, Monash University, Melbourne, VIC 3004, Australia; Centre to Impact AMR, Monash University, Melbourne, VIC 3800, Australia

## Abstract

**Background:**

*Pseudomonas aeruginosa* biofilms limit the efficacy of currently available antibacterial therapies and pose significant clinical challenges. Pseudomonal biofilms are complicated further when other markers of persistence such as mucoid and hypermutable phenotypes are present. There is currently a paucity of data regarding the activity of the newer β-lactam/β-lactamase inhibitor combination ceftolozane/tazobactam against *P. aeruginosa* biofilms.

**Methods:**

We evaluated the efficacy of ceftolozane/tazobactam against clinical *P. aeruginosa* isolates, the laboratory isolate PAO1 and its isogenic *mutS*-deficient hypermutator derivative (PAOMS) grown under static and dynamic biofilm conditions. The clinical isolate collection included strains with mucoid and hypermutable phenotypes.

**Results:**

Ceftolozane/tazobactam exposure led to a bactericidal (≥3 log cfu/cm^2^) biofilm reduction in 15/18 (83%) clinical isolates grown under static conditions, irrespective of carbapenem susceptibility or mucoid phenotype, with greater activity compared with colistin (*P* < 0.05). Dynamically grown biofilms were less susceptible to ceftolozane/tazobactam with active biofilm reduction (≥1 log cfu/cm^2^) observed in 2/3 isolates. Hypermutability did not affect the antibiofilm efficacy of ceftolozane/tazobactam in either static or dynamic conditions when comparing PAO1 and PAOMS. Consistent with the activity of ceftolozane/tazobactam as a potent inhibitor of PBP3, dramatic impacts on *P. aeruginosa* morphology were observed.

**Conclusions:**

Our data demonstrate that ceftolozane/tazobactam has encouraging properties in the treatment of *P. aeruginosa* biofilm infections, and its activity is not diminished against mucoid or hypermutable variants at the timepoints examined.

## Introduction

*Pseudomonas aeruginosa* is an opportunistic pathogen responsible for a range of life-threatening nosocomial infections,^1–3^ including septicaemia, intra-abdominal, urinary tract and burn wound infections.^4–10^ In patients with cystic fibrosis (CF), *P. aeruginosa* is one of the predominant causes of persistent lung infection, leading to reduced lung function and increased rates of morbidity and mortality.^11–13^ Within the CF lung, *P. aeruginosa* strains undergo adaptations allowing them to persist in this hostile environment, such as alginate hyperproduction (resulting in a mucoid phenotype), the emergence of hypermutators, antibiotic resistance development and the transition to a biofilm lifestyle.^14–19^ Pseudomonal biofilms have also been implicated in medical device-related infections, including urinary and central venous catheters, as well as mechanical ventilators.^20^

With standard antibiotic regimens becoming increasingly ineffective in the treatment of antibiotic-resistant isolates, carbapenem-resistant *P. aeruginosa* has been declared by the WHO as high priority, urgently requiring new antibiotics.^21^ Notably, one of the main antibiotic resistance mechanisms of *P. aeruginosa* is the production of AmpC β-lactamase—a class C cephalosporinase—mediating resistance against many β-lactam antibiotics.^22–25^ Other resistance mechanisms include the inactivation of the porin channel OprD, reducing outer membrane permeability to carbapenems and the overexpression of efflux pumps.^24–26^ The transition to a biofilm lifestyle is also associated with persistent *P. aeruginosa* infections in high-risk groups.^18,27^ Biofilms afford already MDR *P. aeruginosa* additional protection from antibiotic therapy, and host immune defences.^28^

Ceftolozane/tazobactam is a β-lactam/β-lactamase inhibitor combination, and a valuable therapeutic option for the treatment of MDR *P. aeruginosa* infections.^29–32^ Ceftolozane/tazobactam is currently approved for the treatment of complicated intra-abdominal and urinary tract infections, as well as hospital- and ventilator-acquired pneumonia.^31^ Ceftolozane is a semisynthetic cephalosporin with structural similarity to ceftazidime. Improvements over ceftazidime include the addition of a bulky pyrazole ring conferring increased stability against AmpC β-lactamase via steric hindrance.^33,34^ Furthermore, the activity of ceftolozane is unaffected by Mex efflux pumps and does not rely on OprD for its transition across the outer membrane.^34^ Similar to other cephalosporins, ceftolozane inhibits cell wall synthesis by binding the PBPs.^35^ Ceftolozane binds to all *P. aeruginosa* essential PBPs (1b, 1c, 2 and 3) with higher affinity than ceftazidime, but retains low PBP4 affinity, making it a weak inducer of AmpC.^35^ The addition of tazobactam broadens the activity of ceftolozane to include ESBL-producing Enterobacterales.^36^ Studies evaluating the efficacy of ceftolozane/tazobactam against clinical *P. aeruginosa* isolates confirmed its superior activity compared with other routinely prescribed antipseudomonal β-lactams (e.g. ceftazidime, piperacillin/tazobactam and cefepime) and good activity against MDR and XDR strains.^37–40^ Resistance to ceftolozane/tazobactam has been reported and is often mediated via mutations that confer structural modifications and overexpression of AmpC and carbapenemase production.^41,42^ To date, there have been a limited number of studies assessing the efficacy of ceftolozane/tazobactam against *P. aeruginosa* biofilms.^43–46^

In the current study, we selected 18 clinical isolates recovered from high-risk patient groups, and a laboratory strain and its isogenic hypermutator derivative to evaluate the efficacy of ceftolozane/tazobactam against *P. aeruginosa* using both static and dynamic biofilm growth conditions. Dynamic biofilms were generated using a CDC Biofilm Reactor (CBR) where continuous flow was exploited to simulate the pharmacokinetic (PK) profile of ceftolozane/tazobactam. To the best of our knowledge, this is the first paper to demonstrate the antibiofilm efficacy of ceftolozane/tazobactam against *P. aeruginosa* isolates with a range of antibiotic resistance and persistence phenotypes in both static and dynamic assays.

## Materials and methods

### Bacterial isolates, media and antibiotics

A total of 18 clinical isolates from patients at The Alfred Hospital, Melbourne, Australia were selected (Table 1 and Table S1, available as Supplementary data at *JAC* Online). All isolates were susceptible to ceftolozane/tazobactam and colistin (EUCAST v14.0),^47^ including mucoid and non-mucoid variants, as well as a range of meropenem MICs (≤0.12 to ≥ 16 mg/L). Ceftolozane/tazobactam, colistin and meropenem MICs were determined by broth microdilution. The reference strain, PAO1, and its isogenic hypermutator derivative (PAOMS) were also included.^48^ Isolates were cultured in CAMHB at 37°C with vigorous shaking. Ceftolozane/tazobactam, provided by Merck as the commercial formulation Zerbaxa™, was prepared according to the manufacturer’s instructions. Stock solutions of colistin sulphate (Sigma–Aldrich) were prepared in sterile water.

**Table 1.** List of isolates used in this study and their characteristics

Strain	Source	Morphology	Meropenem MIC (mg/L)	C/T MIC (mg/L)^a^	Activity against static biofilms (C/T mg/L)^b^			Activity in a PK/PD model at 24 h (C/T 2 g/1 g q8h)^b^	Activity in a PK/PD model at 24 h CST 3.5 mg/L CI	Hypermutability (RIF MF)^c^
					4/2	90/45	140/70			
PAO1	Laboratory strain	Non-mucoid	≤0.5	≤0.5	A	C	C	A	—	—
PAOMS	Laboratory strain	Non-mucoid	≤0.5	≤0.5	A	C	C	A	—	166^d^
MS-1	Sputum	Mucoid	≤0.12	1	C	C	C	—	—	1.73
MS-2	Sputum	Mucoid	≤0.12	1	A	C	C	—	—	4.41
MS-3	Sputum	Non-mucoid	≤0.12	1	C	C	C	—	—	2.14
MS-4	Sputum	Non-mucoid	0.25	≤0.5	A	C	C	—	—	1.15
MS-5	Sputum	Non-mucoid	≤0.12	1	S	C	C	—	—	0.82
MS-6	Sputum	Non-mucoid	≤0.12	≤0.5	A	C	C	A	A	0.55
MS-7	Sputum	Non-mucoid	≤0.12	≤0.5	A	C	C	—	—	1.4
MI-1	Sputum	Mucoid	4	1	A	C	C	—	—	3.25
MI-2	Sputum	Non-mucoid	4	2	A	A	C	S	S	13.94
MI-3	Sputum	Non-mucoid	4	≤0.5	A	A	A	—	—	368.51^d^
MI-4	Sputum	Non-mucoid	4	1	A	A	A	—	—	135.97^d^
MI-5	Sputum	Non-mucoid	4	1	A	C	C	—	—	1.56
MI-6	Sputum	Mucoid	4	2	A	C	C	—	—	7.2
MR-1	Burn swab	Non-mucoid	≥16	2	A	C	C	—	—	5.72
MR-2	Sputum	Non-mucoid	≥16	4	A	C	C	A	S	0.24
MR-3	Sputum	Non-mucoid	≥16	1	A	A	A	—	—	4.7
MR-4	Sputum	Non-mucoid	≥16	≤0.5	A	C	C	—	—	13.61
MR-5	Sputum	Non-mucoid	≥16	≤0.5	A	C	C	—	—	256.42^d^

C/T, ceftolozane/tazobactam; CST, colistin; CI, continuous infusion; RIF, rifampicin; MF mutator frequency. —, this parameter was not determined for this isolate.

^a^The tazobactam concentration was fixed at 4 mg/L.

^b^Biofilm reduction <1 log cfu/cm^2^ was considered bacteriostatic (S),  ≥ 1 log cfu/cm^2^ was considered active (A) and ≥3 log cfu/cm^2^ was considered bactericidal (C).

^c^MFs were calculated as the fraction of resistant bacteria quantified on 300 mg/L RIF-containing media compared with total bacteria quantified on antibiotic-free media. Hypermutators were defined as isolates with rifampicin MFs ≥20-fold higher than PAO1.

^d^Indicates a strain with a hypermutable phenotype.

### Static biofilm assay

Sterile silicone coupons (Biosurface Technologies) were inoculated with ∼10^8^ cfu/mL. Coupons were incubated at 37°C for 90 min with gentle agitation to allow bacterial adherence, and washed three times in PBS to remove non-adherent cells. Biofilms were established by incubating coupons in fresh media for 24 h at 37°C with gentle agitation. Coupons were washed as previously described, and fresh media containing either 4/2, 90/45 or 140/70 mg/L ceftolozane/tazobactam (representative of ceftolozane susceptibility breakpoint and serum *fC*_max_ of low and high doses, respectively),^49^ or 3.5 mg/L colistin (representative of steady-state serum concentration)^50^ was added and incubated at 37°C for 24 h with mild agitation. Coupons were washed in PBS and biofilm density enumerated as previously described to determine cfu/cm^2^.^51^ For samples below the limit of detection, 40 cfu/cm^2^ was used. Experiments were performed in technical triplicate with four biological replicates.

### Dynamic biofilm assay

Biofilms were generated under shear force conditions as previously described, with some modifications.^51^ Briefly, the CBR (Biosurface Technologies) containing 350 mL of CAMHB was inoculated with ∼10^8^ cfu. Eight polypropylene rods, each housing three silicone coupons, were suspended from the lid, for biofilm growth. The isolates underwent 24 h batch phase culture at 37°C with shear force provided by a baffled stirbar operating at 120 rpm. This was followed by a 24 h biofilm conditioning phase where 10% CAMHB was infused at a rate of 11.67 mL/min using a peristaltic pump. Following this was a 24 h therapeutic phase mimicking the serum ceftolozane *f*AUC, *fC*_max_ and %*fT*_>MIC_ reported in patients receiving 2 g/1 g ceftolozane/tazobactam as a 1 h infusion every 8 h: *f*AUC_0–24_ 935 mg·h/L, *fC*_max_ 120 mg/L, protein binding considered 21%, *fT*_>MIC_ 100% (>4 mg/L).^52^ Colistin was supplied as a bolus dose, followed by continuous infusion at 3.5 mg/L, mimicking patient steady-state concentrations, as previously described.^45,53^ Coupons were removed aseptically at 0, 8, 16 and 24 h post antibiotic administration for cfu enumeration and imaging analysis. Bacterial viability was determined from at least three coupons that were washed and prepared as previously described. Each experiment is representative of at least three biological replicates.

### Data analysis

Antimicrobial efficacy was determined by calculating log reductions of viable cells [log_10_ (treated viable cell density/untreated viable cell density)]. Treatments resulting in a <1 log,  ≥ 1 log and ≥3 log cfu/cm^2^ reductions at 24 h were considered bacteriostatic, active and bactericidal, respectively.^45,54^ Comparison of cfu reduction in the static and dynamic biofilm models were made using a one-way ANOVA with a Tukey’s multiple comparisons test. Biofilm density in static versus dynamic models and ceftolozane/tazobactam versus colistin in static assays were analysed using a Mann–Whitney test (GraphPad Prism 9.0.1).

### PK validation

Ceftolozane/tazobactam concentrations in the CBR were determined by Pathology Queensland using standardized methods. Medium from the CBR was sampled at 1, 2, 4, 6, 8 and 24 h following ceftolozane/tazobactam administration, filter sterilized (0.2 μm) and stored at −80°C until analysis. Unbound concentrations were separated using an Amicon Ultra 0.5 mL 30 000-molecular-weight-cutoff centrifugal filter device (Merck Millipore, Sydney, Australia). Ceftolozane concentrations were quantified using a validated UPLC coupled with QDa mass detection (Waters Corporation, Milford, MA, USA). The lower limit of quantification was 0.1 mg/L and the imprecision was <10% at all levels. For colistin measurements, medium was sampled from the CBR at 0 and 24 h and stored at −80°C until analysis. Colistin concentrations were analysed using HPLC coupled with triple quadrupole MS (Shimadzu, Japan). The standard curve of colistin ranged from 0.1 to 10 mg/L and the correlation coefficient was over 0.996. The quality controls (QCs) were at 0.1, 1.0, 4.0 and 8.0 mg/L and the intra- and inter-day precision based on the standard deviation of QC replicates was <10%, with accuracy ranging from 96% to 102%.

### Microscopy

Coupons from the CBR were prepared for scanning electron microscopy (SEM) as previously described.^55^ Images were captured using a Nova NanoSEM. Coupons taken from the CBR at 0 and 24 h were also stained using the LIVE/DEAD™ *Bac*Light™ Bacterial Viability Kit (L7007, Thermo Fisher Scientific) according to the manufacturer’s instructions. Samples were imaged using a Leica SP5 confocal laser scanning microscope (CLSM) with sequential scanning at 488 and 561 nm.

### Hypermutable phenotype

Mutation frequencies to rifampicin (300 mg/L) were determined as previously described.^17^ Assays were performed in technical triplicate, with three biological replicates. Frequencies >20-fold higher than that observed for PAO1 were defined as hypermutators.

## Results

### P. aeruginosa strains

Isolates were recovered from patients with a range of clinical presentations, including a burn wound, infective exacerbation of CF, lung transplant and other chronic lung conditions (Table 1). All strains were susceptible to ceftolozane/tazobactam and colistin in accordance with EUCAST interpretations, with MICs ranging from ≤0.5/4 to 4/4 mg/L, and 0.25 to 2 mg/L, respectively (Table 1). Given the importance of carbapenem-resistant *P. aeruginosa*, the strains were purposely selected to represent a range of meropenem susceptibility phenotypes (MICs ≤0.12 to  ≥ 16 mg/L). Strains with additional persistence phenotypes were identified, including four mucoid and three hypermutable phenotypes (Table 1).

### Activity of ceftolozane/tazobactam against P. aeruginosa in a static biofilm assay

**Figure 1.**
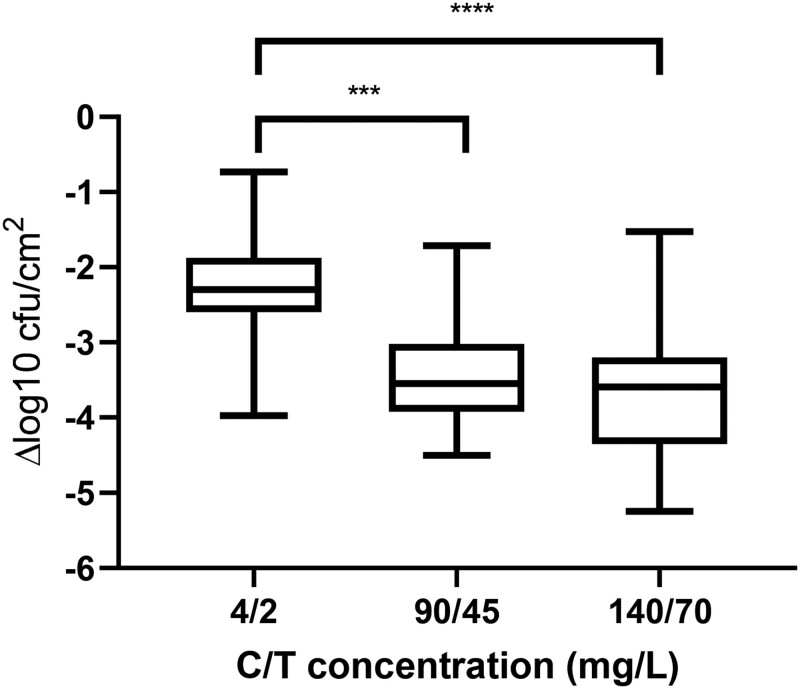
Ceftolozane/tazobactam activity against static *P. aeruginosa* biofilms. Combined biofilm cfu reductions of 18 *P. aeruginosa* clinical isolates and PAO1 following treatment with varying concentrations of ceftolozane/tazobactam, with each isolate tested four times. Results are expressed as log_10_ reduction of cfu/cm^2^ compared with untreated controls. ***P ≤ 0.001, ****P ≤ 0.0001. C/T, ceftolozane/tazobactam.

Ceftolozane/tazobactam efficacy against static biofilms was assessed for 18 *P. aeruginosa* isolates and the reference strain PAO1 (Table 1 and Table S1), using concentrations representative of the ceftolozane MIC breakpoint (4 mg/L) and two clinically relevant concentrations mimicking the *fC*_max_ of standard and high doses (90/45 and 140/70 mg/L, respectively).^49^ All isolates formed biofilms under static conditions (Figure S1). Ceftolozane/tazobactam was active against 15/18 clinical isolates and PAO1 at 4/2 mg/L, with bacteriostatic and bactericidal activity observed against one and two isolates, respectively, at this concentration (Table 1). At 90/45 and 140/70 mg/L, bactericidal activity was observed against 14/18 and 15/18 isolates, respectively, with active reduction observed in the remaining isolates (Table 1, Figure S2). Ceftolozane/tazobactam was bactericidal against PAO1 at these concentrations. Overall reductions in biofilm cfu were observed at all concentrations tested, with significant decreases observed at 90/45 (*P* < 0.001) and 140/70 mg/L (*P* < 0.0001) compared with 4/2 mg/L (Figure 1). No significant difference was observed between 90/45 and 140/70 mg/L (Figure 1).

Ceftolozane/tazobactam displayed good activity against clinical isolates, regardless of meropenem susceptibility and mucoid phenotypes (Figure S2). The ceftolozane/tazobactam MIC (when grown in the planktonic state) was not a good predictor of antibiofilm efficacy. Isolates with the highest ceftolozane/tazobactam MICs (2/4 to 4/4 mg/L) demonstrated a >3 log reduction, while three isolates that failed to reach the bactericidal criteria effect had a lower MIC range (≤0.5/4 to 1/4 mg/L) (Table 1). Additionally, ceftolozane/tazobactam demonstrated bactericidal activity against three ceftazidime-resistant isolates (MI-2, MI-6 and MR-1). We also compared the antibiofilm activity of ceftolozane/tazobactam with that of colistin for three randomly selected clinical isolates, one from each meropenem susceptibility group (MS-6, MI-2, MR-2). Colistin demonstrated significantly lower efficacy against static biofilms when compared with 140/70 mg/L ceftolozane/tazobactam (*P* < 0.05) (Figure S3).

### Activity of ceftolozane/tazobactam against P. aeruginosa using a dynamic biofilm assay

**Figure 2.**
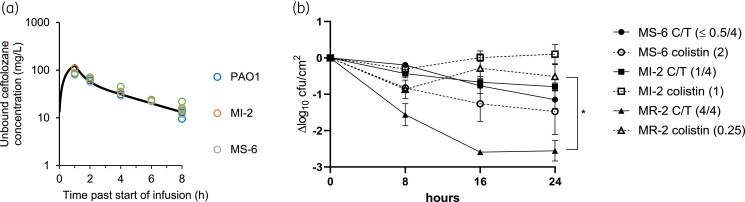
Ceftolozane/tazobactam activity against *P. aeruginosa* biofilms in a PD model. (a) PK validation of the PD biofilm assay for ceftolozane/tazobactam. The 24 h sample is representative of 8 h post infusion and is represented together with the 8 h result in this position. (b) Bacterial killing by ceftolozane/tazobactam and colistin over 24 h in a PD biofilm assay. Ceftolozane/tazobactam was infused into the reactor over 1 h via a syringe pump at 0, 8 and 16 h. Colistin was supplied as a continuous infusion. Biofilm-embedded coupons were sampled at 0, 8, 16 and 24 h. Results are expressed as log_10_ reduction of cfu/cm^2^ compared with time 0 (mean ± standard error of the mean). The numbers in brackets indicate the antibiotic MICs for each isolate in mg/L. Each point is representative of three (colistin) or four (ceftolozane/tazobactam) biological replicates. Ceftolozane/tazobactam demonstrated significantly greater efficacy than colistin against isolate MR-2 (*P* < 0.05, one-way ANOVA). C/T, ceftolozane/tazobactam.

Antibiofilm activity of ceftolozane/tazobactam at concentrations mimicking 2/1 g q8h for 24 h was investigated for isolates MS-6, MI-2 and MR-2 using the CBR. To validate the ceftolozane PK, the concentration was measured at various intervals over 24 h. Concentration–time profiles observed in our assay corresponded to those achieved in patients based on a previously published PK model (Figure 2a).^52^ Biofilm formation was confirmed for all isolates at the conclusion of the conditioning phase, but prior to ceftolozane/tazobactam infusion (T0), with most forming denser biofilms than their statically grown counterparts (Figure S4). Viable bacteria were enumerated at 8 (T8), 16 (T16) and 24 h (T24) following ceftolozane/tazobactam exposure and compared with T0 to determine the log reduction (cfu/cm^2^) (Figure 2b). The greatest log reduction at T24 was observed for isolate MR-2 (2.6 log cfu/cm^2^), followed by MS-6 (1.2 log cfu/cm^2^) and MI-2 (0.8 log cfu/cm^2^) (Figure 2b). The overall susceptibility of strains in the CBR was reduced compared with the static biofilm model (at 90/45 and 140/70 mg/L), with ceftolozane/tazobactam bacteriostatic against 1/3 isolates and active against 2/3 isolates tested (Table 1).

Despite failing to meet the criteria for bactericidal activity, the log reductions observed correlate with 99.6%, 93.3% and 86.9% killing of the bacterial population for MR-2, MS-6 and MI-2, respectively, at T24. Although MR-2 had the highest ceftolozane/tazobactam MIC, its biofilm was the most susceptible to ceftolozane/tazobactam (Figure 2b). Despite forming the least dense biofilm at T0, MI-2 was the least susceptible to killing by ceftolozane/tazobactam (Figure 2a and Figure S4).

For comparison, the activity of colistin was assessed against the same isolates. To validate the colistin PK in our assay, the concentration was measured at the beginning and end of the treatment phase. The concentration range and median (range 0.725–3.95 mg/L, median 2.57 mg/L) were slightly below our target median of 3.5 mg/L but were consistent with the flat plasma concentration–time profiles of formed colistin previously reported in patients.^50^ The greatest reduction in bacterial viability at T24 was observed in isolate MS-6 (1.4 log cfu/cm^2^), similar to that of ceftolozane/tazobactam (1.2 log cfu/cm^2^). This was despite MS-6 forming the densest biofilm at T0 and having the highest colistin MIC (2 mg/L). In contrast, colistin had poor efficacy against the other isolates (Figure 2b). Biofilms in untreated CBRs remained stable over the 24 h duration (Figure S5).

### Ceftolozane/tazobactam exposure alters cell morphology

**Figure 3.**
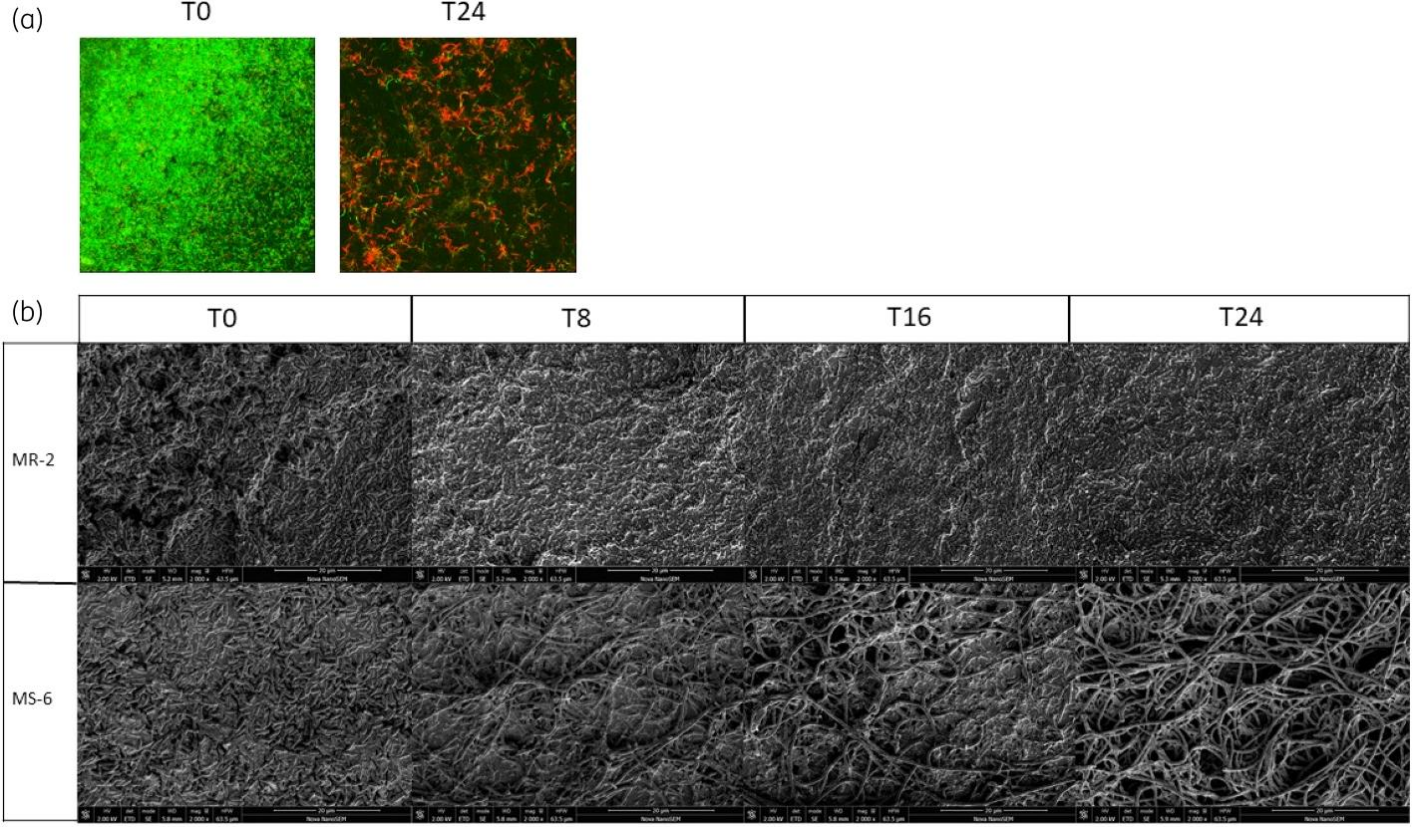
Effect of ceftolozane/tazobactam treatment on biofilm integrity. (a) Representative CLSM images of LIVE/DEAD stained biofilm embedded cells at 0 and 24 h following ceftolozane/tazobactam treatment in the CBR. Live cells appear green and dead cells appear red. At 0 h there is a dense biofilm present consisting of live cells. By 24 h the biofilm is sparser with many dead cells and altered cell morphology apparent. Images shown are of strain PAO1. (b) Representative SEM images showing the range of cellular morphologies observed following ceftolozane/tazobactam treatment. All isolates have dense biofilms prior to treatment. From 8 h onwards, MR-2 displays a loss of cellular integrity and presence of cellular debris (phenotype 1). From 8 h onwards, MS-6, MI-2, PAO1 and PAOMS display filamentation (phenotype 2) consistent with potent inhibition of PBP3.

Following ceftolozane/tazobactam treatment, biofilm appearance and cell morphology were assessed by microscopy. Prior to antibiotic treatment, well-defined biofilms were observed with CLSM, while at 24 h post treatment these were sparser and predominantly consisted of dead cells, with significantly altered morphology (Figure 3a). Cell morphology was examined further using SEM. At T0, varying degrees of biofilm density, surface irregularity and architectural complexity were observed. Filament formation was observed in isolates MS-6 and MI-2 by T8, and was more advanced at T16 and T24. Isolate MR-2 demonstrated a loss of cellular integrity with cellular debris observed by T8 that was considerably more pronounced at T16 and T24 (Figure 3b).

### Activity of ceftolozane/tazobactam against P. aeruginosa hypermutators

As hypermutable strains of *P. aeruginosa* are often linked to treatment failure, we compared the efficacy of ceftolozane/tazobactam against PAO1 and its isogenic *mutS*-deficient hypermutator derivative PAOMS.^48^ Similar biofilm densities were observed for both strains in the static and dynamic assays (Figure S4), with similar reductions observed post ceftolozane/tazobactam treatment. In the static assay, 4/2 mg/L ceftolozane/tazobactam was active against both strains, while treatment with either 90/45 or 140/70 mg/L was bactericidal (Figure S6a). In the CBR, PAOMS had a 1.9 log cfu reduction in biofilm bacterial density at T24 (Figure S6b), equal to that observed for PAO1. The morphological impact of ceftolozane/tazobactam on PAOMS was similar to that observed for PAO1 by CLSM and SEM (Figure 3).

Following susceptibility testing, we determined the hypermutator capability of our clinical isolates. Three isolates (MI-3, MI-4 and MR-5) were identified as hypermutator strains, with a 136-fold to 369-fold increase in mutation frequency compared with PAO1 (Table 1), consistent with the 166-fold increased mutation frequency observed for PAOMS. Of the three hypermutator isolates identified, ceftolozane/tazobactam was active against MR-5 under static biofilm conditions at 4/2 mg/L and bactericidal at 90/45 (3.7 log reduction) and 140/70 mg/L (5.3 log reduction) (Figure S6a). Ceftolozane/tazobactam was active against isolates MI-3 and MI-4 at all concentrations tested (Figure S6a).

## Discussion

In the present study, we evaluated the efficacy of ceftolozane/tazobactam against *P. aeruginosa* biofilms under static and dynamic conditions. Ceftolozane/tazobactam demonstrated more pronounced antibiofilm activity than our comparator, colistin, and was unaffected by MIC, mucoid or hypermutator phenotypes. While more pronounced activity was observed under static compared with dynamic conditions, the exact reasons for this are currently unclear. While previous studies have presented conflicting findings regarding the efficacy of ceftolozane/tazobactam against *P. aeruginosa* biofilms,^43–45^ these discrepancies may be related to differences in the method of biofilm generation, media, isolate selection and endpoint. In contrast to statically grown biofilms, those grown under dynamic conditions are exposed to shear stress, undergo media replacement and are amenable to PK/pharmacodynamic (PD) modelling, impacting biofilm density and response to antibiotic treatment. Consistent with that reported elsewhere, biofilms grown in dynamic flow systems are known to be less susceptible to treatment compared with their statically grown counterparts.^54,56^

In the limited *in vitro* studies investigating ceftolozane/tazobactam efficacy against pseudomonal biofilms, inconsistent media types were used.^43–45,46^ In the current study we selected CAMHB, due to its low protein-binding properties, allowing for accurate PK profiling. Interestingly, staphylococcal and pseudomonal biofilms formed in TSB media are less susceptible to antibiotic treatment than those formed in CAMHB,^57^ and while similar observations have been reported for ceftolozane/tazobactam-treated pseudomonal biofilms, a direct comparison is not possible due to different assay parameters and endpoints.^43,44^ Similarly, while Gomez-Junyent *et al*.^45^ reported weak ceftolozane/tazobactam activity against pseudomonal biofilms in TSB, as compared with the current study, the former also utilized a *fC*_max_ of 90 mg/L compared with 120 mg/L in the current study. Therefore, when interpreting susceptibility data, it is important to consider the impact of media and biofilm assay type (static or dynamic).

We observed that the ceftolozane/tazobactam MIC was not a predictor of antibiofilm efficacy. It’s possible that differences in polysaccharide composition, extracellular appendages and quorum-sensing molecules may affect biofilm structure, influencing antibiotic binding, penetration and effectiveness.^58,59^ Further investigations into these phenotypes may provide insights into the differences observed between isolates. Furthermore, biofilm density has also been implicated in variations of drug susceptibility;^60^ however, this was not a contributing factor in the current study, with no correlation between density and killing observed. Finally, despite concentration-independent bactericidal activity at clinically relevant doses, the site of infection should determine the dosing regimen. In humans, a 1.5 g dosing regimen (q8h) is recommended for complicated urinary tract and intra-abdominal infections.^61^ However, as the plasma to epithelial lining fluid (ELF) ratio is 50%, double dosing regimens of 3 g are recommended for the treatment of hospital- and ventilator-acquired pneumonia.^61^ This achieves higher exposure with a PTA of >90% in the ELF.^61,62^

Consistent with the known mechanism of action of ceftolozane/tazobactam, significant morphological changes were observed for all isolates following treatment, with drastic filamentation observed, likely as a result of PBP3 inhibition.^35^ Interestingly, striking differences were observed over time with the loss of cellular integrity and increasing debris at the later timepoints, which was more severe in isolates with the greatest biofilm reduction. We hypothesize this marked difference in morphology may impact bacterial physiology and fitness similar to that observed in pathogens with *pbp* mutations.^63,64^

As hypermutator *P. aeruginosa* strains are associated with antibiotic resistance and poorer outcomes,^15^ we sought to confirm whether this capacity affects ceftolozane/tazobactam efficacy. Consistent with that reported previously,^43^ we found that hypermutation did not impact ceftolozane/tazobactam efficacy; however, these experiments represent short antibiotic exposure time frames. Of the three clinical hypermutators tested in this study, two were the least susceptible to ceftolozane/tazobactam, though the drug was still active, while the third was one of the most susceptible isolates, with the second greatest biofilm reduction. Given that no difference in the ceftolozane/tazobactam efficacy was observed against PAO1 and its isogenic hypermutator PAOMS, it is more likely that matrix composition and biofilm structure affects ceftolozane/tazobactam efficacy rather than hypermutability in this study. Studies investigating long-term exposure of PAOMS to ceftolozane/tazobactam reported high-level resistance, mediated by multiple mutations leading to AmpC structural modification and overexpression.^41^ Nevertheless, resistance developed at a slower rate compared with other antipseudomonal agents and was associated with increased susceptibility to penicillins and carbapenems. It has therefore been suggested that combination therapy may be required to suppress the emergence of resistance.^46,65^

Our study identified several aspects requiring additional research. Firstly, matrix composition, which plays an important role in antibiotic activity, was not examined and is beyond the scope of this study. Secondly, the 24 h treatment duration is shorter than most standard regimens, and it remains to be determined whether longer treatment phases would yield different results or lead to the emergence of resistance. Mucoid phenotypes and hypermutable capacity emphasize the need for further investigations, particularly over longer exposures. Thirdly, the emergence of resistance was not addressed in our models. This is a notable consideration as the *in vitro* development of ceftolozane/tazobactam resistance has previously been reported, particularly for hypermutable isolates.^41,46,65^ Lastly, the experimental conditions and growth surface used in this study mimic device-related biofilm infections but do not replicate the conditions found in patients with invasive infections.

In summary, ceftolozane/tazobactam has activity against infections caused by non-carbapenemase-producing *P. aeruginosa* where the success of current therapies is limited due to the presence of highly resistant biofilms, regardless of meropenem susceptibility, colony morphotype and hypermutability.

## Supplementary Material

dkae413_Supplementary_Data

## Supplementary data

Figures S1 to S6 and Table S1 are available as Supplementary data at *JAC* Online.
