# Supplementary material for: Ceftolozane/tazobactam disrupts Pseudomonas aeruginosa biofilms under static and dynamic conditions
Source: J Antimicrob Chemother. 2024 Dec 9;80(2):372–80. doi: 10.1093/jac/dkae413 (PMC11787898; doi:10.1093/jac/dkae413)
Supplement: dkae413_Supplementary_Data [file dkae413_supplementary_data.docx]

**Table S1:** Antibiotic susceptibility of clinical isolates according to EUCAST v14.0

| Strain | Clinical interpretation | | | | | | |
| --- | --- | --- | --- | --- | --- | --- | --- |
|  | MEM | AMK | CAZ | CIP | TOB | TZP^‡^ | CST^‡^ |
| MS-1 | S (≤0.12) | S (32) | I (1) | R (2) | S (1) | I (1) | S (1) |
| MS-2 | S (≤0.12) | S (4) | I (1) | R (2) | S (1) | I (1) | S (1) |
| MS-3 | S (≤0.12) | S (8) | I (1) | I (0.5) | S (1) | I (1) | S (1) |
| MS-4 | S (0.25) | S (8) | I (1) | I (0.5) | S (1) | I (1) | S (1) |
| MS-5 | S (≤0.12) | S (8) | I (1) | R (2) | S (1) | I (1) | S (0.5) |
| MS-6 | S (≤0.12) | S (4) | I (2) | I (0.12) | S (1) | I (4) | S (2) |
| MS-7 | S (≤0.12) | S (4) | I (0.5) | I (0.06) | S (1) | I (1) | S (1) |
| MI-1 | I (4) | S (8) | I (8) | R (1) | S (1) | R (32) | S (0.5) |
| MI-2 | I (4) | S (4) | R (16) | R (2) | R (8) | R (>32) | S (1) |
| MI-3 | I (4) | S (8) | I (0.5) | R (2) | S (2) | I (1) | S (1) |
| MI-4 | I (4) | S (8) | I (0.5) | R (2) | S (1) | I (1) | S (1) |
| MI-5 | I (4) | S (8) | I (4) | I (0.5) | S (1) | I (16) | S (1) |
| MI-6 | I (4) | S (8) | R (16) | R (2) | S (1) | R (>32) | S (0.5) |
| MR-1 | R (≥16) | S (4) | R (16) | I (0.5) | S (1) | R (>32) | S (2) |
| MR-2 | R (≥16) | R (32) | I (4) | R (2) | R (8) | R (>32) | S (0.25) |
| MR-3 | R (≥16) | R (32) | I (1) | R (1) | S (2) | I (1) | S (1) |
| MR-4 | R (≥16) | S (4) | I (4) | I (0.5) | S (1) | I (8) | S (1) |
| MR-5 | R (≥16) | S (4) | I (2) | R (2) | S (1) | I (4) | S (0.5) |

MICs had been previously determined by broth microdilution or Vitek. Susceptibility is reported in accordance with EUCAST v14.0 breakpoints. S = susceptible, I = susceptible, increased exposure, R = resistant. MEM = meropenem, AMK = amikacin, CAZ = ceftazidime, CIP = ciprofloxacin, TOB = tobramycin, TZP = piperacillin-tazobactam, CST = colistin. ^‡^ The tazobactam concentration was fixed at 4 mg/L. Brackets indicate MICs in mg/L.

^‡^ These isolates are considered intermediate per CLSI guidelines.

**Figure S1: Biofilm density of *P. aeruginosa* isolates on silicone coupons in a static assay.** Each bar represents the mean of four replicates. Error bars indicate the standard error of mean**.**

**Figure S2: Ceftolozane/tazobactam activity against static biofilms.** Antibiofilm efficacy of ceftolozane/tazobactam against (A) meropenem susceptible (MIC ≤ 2 mg/L), (B) meropenem susceptible, increased exposure (MIC = 4 mg/L), and (C) meropenem resistant isolates (MIC ≥ 8 mg/L). Results are expressed as log_10_ reduction of cfu/cm^2^ compared to untreated controls. Points below the dashed horizontal line indicate a bactericidal effect of ceftolozane/tazobactam treatment. Data displayed indicate the mean of four replicates ± standard error of mean. (M) = mucoid. C/T = ceftolozane/tazobactam.

**Figure S3: Comparison of ceftolozane/tazobactam and colistin activity against static biofilms** Ceftolozane/tazobactam (140/70 mg/L) has significantly greater efficacy against static biofilms when compared to colistin (3.5 mg/L) against a subset of clinical *P. aeruginosa* isolates (*p* < 0.05). Solid shapes indicate ceftolozane/tazobactam treatment while open shapes indicate biofilms treated with colistin.

**Figure S4: Untreated *P. aeruginosa* biofilm density in static vs dynamic model** Comparison of untreated biofilm formed in either a static model or CBR. Each column is representative of four biological replicates (mean ± standard error of mean). Isolates MS-6, PAO1 and PAOMS formed significantly more biofilm in the dynamic model than in the static model. * = *p* < 0.05.

**Figure S5:** **Biofilm stability in a control CBR without antibiotics.** Biofilm stability was measured by replicating the PK/PD CBR model in the absence of antibiotic treatment.

**Figure S6: Ceftolozane/tazobactam activity against hypermutable strain biofilms.** (A) Antibiofilm efficacy of ceftolozane/tazobactam against PAO1 and hypermutable *P. aeruginosa* isolates in a static biofilm assay. (B) Comparison of PAO1 and PAOMS bacterial killing by ceftolozane/tazobactam over a 24-hr period in a CBR. Results are expressed as log_10_ reduction of cfu/cm^2^ compared to time 0 (mean ± standard error of the mean). Each point is representative of four biological replicates. C/T = ceftolozane/tazobactam.

**Figure 1: Biofilm density of *P. aeruginosa* isolates on silicone coupons in a static assay.** Each bar represents the mean of four replicates. Error bars indicate the standard error of mean.

**A.**

**B.**

**C.**

**Figure S2: C/T activity against static biofilms** Antibiofilm efficacy of ceftolozane/tazobactam against (A) meropenem susceptible (MIC ≤ 2 mg/L), (B) meropenem susceptible, increased exposure (MIC = 4 mg/L), and (C) meropenem resistant isolates (MIC ≥ 8 mg/L). Results are expressed as log_10_ reduction of cfu/cm^2^ compared to untreated controls. Points below the dashed horizontal line indicate a bactericidal effect of ceftolozane/tazobactam treatment. Data displayed indicate the mean of four replicates ± standard error of mean. (M) = mucoid. C/T = ceftolozane/tazobactam.

**Figure S3: Comparison of ceftolozane/tazobactam and colistin activity against static biofilms** Ceftolozane/tazobactam (140/70 mg/L) has significantly greater efficacy against static biofilms when compared to colistin (3.5 mg/L) against a subset of clinical *P. aeruginosa* isolates. Solid shapes indicate ceftolozane/tazobactam treatment while open shapes indicate biofilms treated with colistin.

Data displayed indicate the mean of four replicates ± standard error of mean. * = *p* < 0.05.


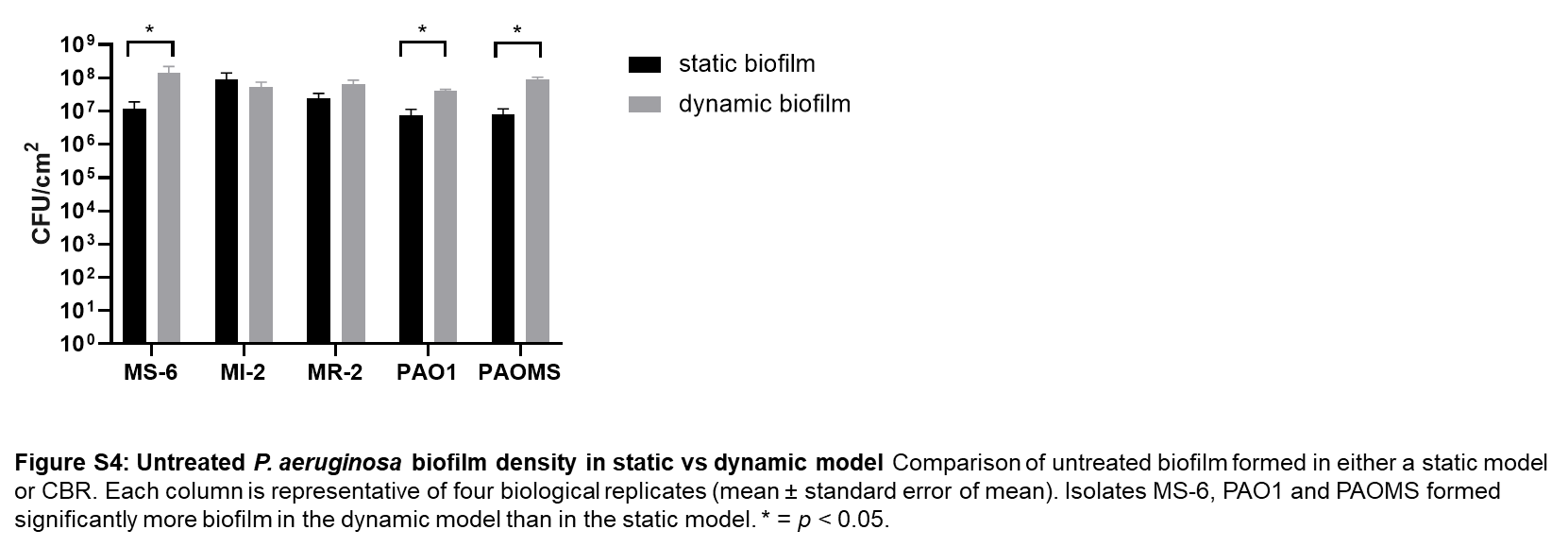


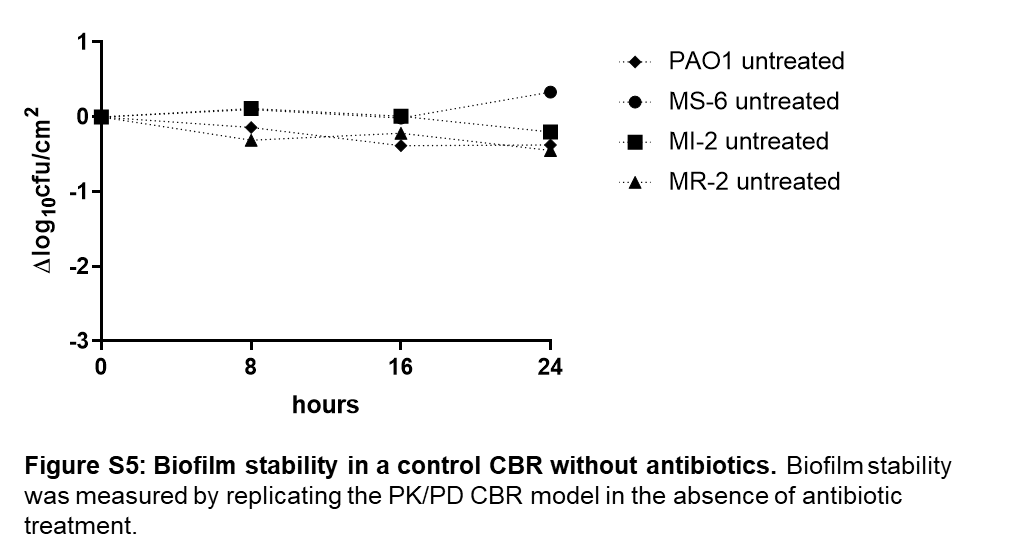


**A.**

**B.**

**Figure S6: Ceftolozane/tazobactam activity against hypermutable strain biofilms.** (A) Antibiofilm efficacy of ceftolozane/tazobactam against PAO1 and hypermutable *P. aeruginosa* isolates in a static biofilm assay. (B) Comparison of PAO1 and PAOMS bacterial killing by ceftolozane/tazobactam over a 24-hr period in a CBR. Results are expressed as log_10_ reduction of cfu/cm^2^ compared to time 0 (mean ± standard error of the mean). Each point is representative of four biological replicates. C/T = ceftolozane/tazobactam.
